# Compound* Schisandra-Ginseng-Notoginseng-Lycium* Extract Ameliorates Scopolamine-Induced Learning and Memory Disorders in Mice

**DOI:** 10.1155/2017/8632016

**Published:** 2017-07-26

**Authors:** Ning Li, Cong Liu, Shu Jing, Mengyang Wang, Han Wang, Jinghui Sun, Chunmei Wang, Jianguang Chen, He Li

**Affiliations:** ^1^College of Pharmacy, Beihua University, Jilin 132013, China; ^2^Affiliated Hospital of Beihua University, Jilin 132013, China

## Abstract

*Schisandra*,* Ginseng*,* Notoginseng*, and* Lycium barbarum* are traditional Chinese medicinal plants sharing cognitive-enhancing properties. To design a functional food to improve memory, we prepared a compound* Schisandra-Ginseng-Notoginseng-Lycium* (CSGNL) extract and investigated its effect on scopolamine-induced learning and memory loss in mice. To optimize the dose ratios of the four herbal extracts in CSGNL, orthogonal experiments were performed. Mice were administered CSGNL by gavage once a day for 30 days and then mouse learning and memory were evaluated by Morris water maze and step-through tests. The mechanisms of CSGNL improving learning and memory were investigated by assaying acetylcholine (ACh) levels and choline acetyltransferase (ChAT) and acetylcholinesterase (AChE) activities in the brain tissues of treated mice. The results showed that CSGNL significantly ameliorated scopolamine-induced learning and memory impairment, at least in part, by modulating ACh levels and ChAT and AChE activities in the mouse brain. Our data support the use of CSGNL as a functional food for learning and memory enhancement.

## 1. Introduction

Learning and memory often decline with age [1]. As life expectancies increase, the number of people suffering from aging-related diseases increases, including learning and memory disorders. Early prevention and treatment may improve the quality of life of affected individuals. Since the neuropathological changes of progressive memory loss and cognitive deficit have been linked to oxidative damage and cholinergic dysfunction [2, 3], free radical scavengers and acetylcholinesterase (AChE) inhibitors have been commonly used to treat these conditions. However, clinical use of these drugs was rather limited due to their excessive side effects and/or high costs [4]. In this regard, traditional herbal medicines are valuable alternatives with the capability of improving learning and memory with fewer side effects.

The dried ripe fruit of a Magnoliaceae plant* Schisandra chinensis* is believed to have beneficial effects, such as tranquilizing the mind, relieving cough and asthma, improving kidney function, and protecting the liver [5]. So far, over 200 compounds have been isolated and identified from* Schisandra*, of which* Schisandra* lignans have been widely used [4].* Schisandra* has been demonstrated to modulate multiple body systems and has an antiaging property. In the central nervous system,* Schisandra* was found to improve learning and memory and exerts sedative and hypnotic effects [6].* Ginseng*, a perennial herb of the Araliaceae family, was thought to excite the central nervous system and reduce fatigue [7]. An active component of* Ginseng*,* ginsenoside*, has been reported to have an antidementia effect by enhancing memory and cognition [8].* Notoginseng* is an* Araliaceae Panax* plant containing the bioactive saponins. Ginsenoside Rg1, ginsenoside Rb1, and notoginsenoside R1 have similar antioxidant properties as well as promoting sedation and intelligence and ameliorating the effects of aging. The dried fruit of* L. barbarum* (Chinese wolfberry) has medicinal benefits, and its main active components,* L. barbarum* polysaccharides, have been reported to modulate immunity, hematopoiesis, and cell activity and to improve learning and memory in an animal model of D-galactose and sodium nitrite-induced aging and to enhance cognition in patients with Alzheimer's disease [9].

In order to develop a functional food capable of improving memory, here we selected extracts from raw materials of* Schisandra, Ginseng, Notoginseng, *and* L. barbarum* and combined them into an herbal formula based on their herbal compatibility. According to the principles of traditional Chinese medicine,* Schisandra* as the monarch drug can replenish Qi, astringe Yin, and nourish the kidney.* Ginseng* and* Notoginseng* as the ministerial drugs invigorate Qi and* Alpinia oxyphylla* for activating blood and dissipate blood stasis.* L. barbarum* as the adjuvant drug can nourish the blood and tonify kidney. We prepared a compound* Schisandra-Ginseng-Notoginseng-Lycium* (CSGNL) and testified its effects on scopolamine-induced learning and memory loss in mice. We also investigated the underlying mechanism by measuring acetylcholine (ACh) levels and choline acetyltransferase (ChAT) and AChE activities in the brain tissues of scopolamine-treated mice. To the best of our knowledge, this is the first study on evaluation of the memory-enhancing effect of CSGNL.

## 2. Materials and Methods

### 2.1. Animals

Male ICR mice, weighing 20 ± 2 g, were obtained from the Experimental Animal Research Center, Jilin University. The mice were raised at 22–25°C in a 12 h light-dark cycle and with access to food and water ad lib. Animal care and experimental procedures were approved by the Ethics Committee of Jilin University.

### 2.2. Preparation of Individual Herbal Extracts

*Schisandra*,* Ginseng*,* Notoginseng*, and* L. barbarum* raw materials were purchased from Jilin Jiuxin Pharmaceutical Group Co., Ltd. (Jilin, China). Based on the People's Republic of China Pharmacopoeia, 40 g of dry raw materials of each herb was used for extract preparation by heating reflux extraction (Table 1).

In short, ground powders of* Schisandra, Ginseng,* and* Notoginseng* materials were dissolved in an appropriate amount of ethanol. Following extraction, the extracts were concentrated and dissolved in 50 mL of methanol. Diluted extracts were used for later analysis of active components. For* L. barbarum*, the extraction residues were concentrated to 50 mL followed by sequentially mixing of 1 mL with 1 mL H_2_O, 1 mL 5% phenol, and 5 mL sulfuric acid. The mixture was incubated in a water bath at 40°C for 15 min and then cooled to room temperature for the determination of* L. barbarum *polysaccharides.

### 2.3. HPLC Analysis of Active Components in Individual Herbal Extracts

The active components of four herbal extracts were analyzed by high performance liquid chromatography (HPLC; Shimadzu Company, Japan) with an Agilent ZORBAX 300SB-C18 chromatographic column. For schisandrin assay, the mobile phase was a 65% methanol-water system at a flow rate of 1 mL·min^−1^. The detection wavelength was 250 nm and the column temperature was 30°C. For phytochemical analysis of ginsenoside Re, ginsenoside Rg1, and ginsenoside Rb1 in* Ginseng* extract, an acetonitrile-water system was used as the mobile phase (Table 2) at a flow rate of 1 mL·min^−1^. The detection wavelength was 203 nm and the column temperature was 50°C. For the analysis of notoginsenoside R1, ginsenoside Rg1, and ginsenoside Rb1 in* Notoginseng *extract, the acetonitrile-water system (Table 3) was used as the mobile phase at a flow rate of 1 mL·min^−1^. The detection wavelength was 203 nm and the column temperature was 30°C.

Reference chemicals (schizandrin, notoginsenoside, and ginsenoside) obtained from Chengdu Pufei De Biotech were dissolved in methanol at 0.1–0.9 mg·mL^−1^ with a good linear relationship. For schisandrin, the regression equation was *y* = 2*∗*10^7^*x* + 2*∗*10^6^, *R*^2^ = 0.999. For the ginsenosides, the regression equations were *y* = 2.81*∗*10^6^*x* + 1.35*∗*10^4^, *R*^2^ = 0.99962 (ginsenoside Re), *y* = 2.64*∗*10^6^*x* + 5.79*∗*10^3^, *R*^2^ = 0.99991 (ginsenoside Rg1), and *y* = 2.47*∗*10^6^*x* + 3.01*∗*10^3^, *R*^2^ = 0.99963 (ginsenoside Rb1). For the notoginsenosides, the regression equations were *y* = 3*∗*10^6^*x* + 60.45, *R*^2^ = 0.999 (ginsenoside R1), *y* = 3*∗*10^6^*x* + 6717, *R*^2^ = 0.999 (ginsenoside Rg1), and *y* = 2*∗*10^6^*x* + 3797, *R*^2^ = 1 (ginsenoside Rb1), where *x* is the concentration of the reference compounds and *R*^2^ is the coefficient of determination.

### 2.4. UV Spectrophotometry of* L. barbarum* Polysaccharides

Polysaccharides in the* L. barbarum* extract were detected by ultraviolet spectrophotometry with glucose as a reference chemical and 1% aluminum chloride as the color reagent. The detection wavelength was 413 nm. Glucose was prepared at 0.2–1.0 mg·mL^−1^. The linear formula of the glucose standard curve was *y* = 0.8098*x* + 0.0121, *R*^2^ = 0.9974.

### 2.5. Optimal Dosing Ratios of CSGNL Determined by Orthogonal Experiments

We used orthogonal experiments to optimize the combination of four herbal extracts with four factors (herbal extracts) and two levels (doses) each. In the orthogonal experiment, two doses for each herbal extract were set in accordance with the optimum human administration dosage proposed in the Pharmacopoeia of the People's Republic of China (2015). For example, the optimal human administration dosage of* Schisandra* proposed in the pharmacopoeia is 2–6 g·day^−1^; according to the average human body weight of 60 kg, the dosage for human is 0.03–0.1 g·kg^−1^. China Food and Drug Administration requires that, in the experiments of health food functions, the administration dose for mice must be set at 10 times the dose for a human adult. Therefore, we chose the dose of crude materials of* Schisandra* for mice at 0.3–1 g·kg^−1^. For herbal extract, we used the doses of 0.03–0.1 g·kg^−1^ in mouse study based on the extraction efficiency of* Schisandra.* Similarly, two levels of* Ginseng* extract were 0.5–1.5 g·kg^−1^,* Notoginseng* extract 0.16–0.5 g·kg^−1^, and* L. barbarum* extract 1-2 g·kg^−1^.

**Figure 1:**
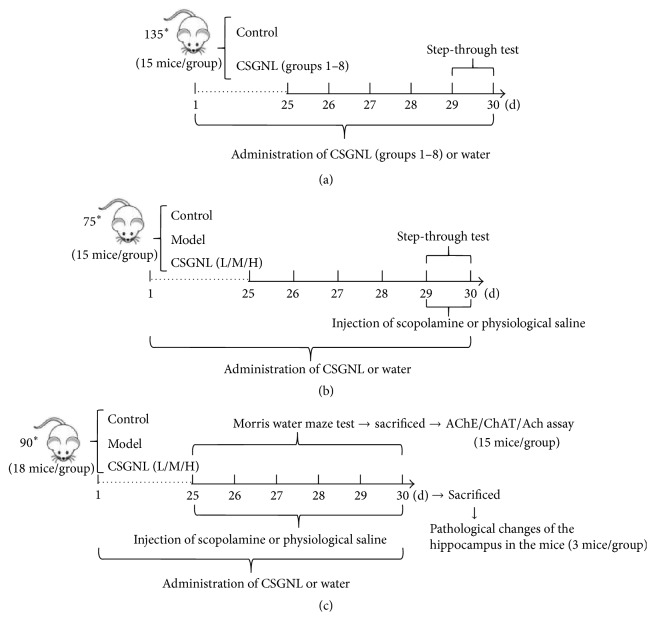
(a) Total 135 mice were randomly divided into nine groups (*n* = 15 each), including one control group administered distilled water and eight treatment groups administered different doses of CSGNL. Mice were administered by gavage once a day 0.1 mL/10 g for 30 days. On days 29 and 30, the step-through test was performed to evaluate the effects of CSGNL dosing ratios. (b) After the optimal dose ratio of CSGNL was determined, mice were divided into five groups of 15 mice each. Group 1 was the control group, and groups 2–5 were administered scopolamine. Groups 1 (control) and 2 (model) received ddH_2_O intragastrically. Groups 3–5 were administered by gavage 0.3 CSGNL-L, 0.6 CSGNL-M, and 1.2 g·kg^−1^ CSGNL-H, respectively, once daily for 30 consecutive days. On days 29 and 30, the step-through test was performed. Ten minutes before the behavioral tests, mice in group 1 were administered normal saline intraperitoneally, and mice in other groups were administered 5 mg·kg^−1^ scopolamine hydrobromide intraperitoneally to induce memory impairment. (c) Mice were divided into five groups of 18 mice each. Group 1 was the control group, and groups 2–5 were administered scopolamine. Groups 1 (control) and 2 (model) received ddH_2_O intragastrically. Groups 3–5 were administered by gavage 0.3 CSGNL-L, 0.6 CSGNL-M, and 1.2 g·kg^−1^ CSGNL-H, respectively, once daily for 30 consecutive days. Ten minutes before the behavioral tests, mice in group 1 were administered normal saline intraperitoneally, and mice in other groups were administered scopolamine. In each group three mice were used for H&E staining and 15 mice for Morris water maze test as well as AChE, ChAT, and ACh assays. *∗*represents the meaning of multiplication.

Total 135 mice were randomly divided into 9 groups (*n* = 15 each), including one control group administered distilled water and eight treatment groups administered different doses of CSGNL. The four herbal extracts were diluted in distilled water (Table 4) at two concentration levels and mixed accordingly for the orthogonal experiments (Table 5). Mice were administered by gavage once a day 0.1 mL·10 g^−1^ for 30 days. On days 29 and 30, the step-through test was performed to evaluate the effects of different CSGNL dosing ratios (Figure 1(a)).

### 2.6. CSGNL Administration

#### 2.6.1. Mouse Grouping for Step-Through Test

After the optimal dose ratio of CSGNL was determined, seventy-five mice were divided into five groups with 15 mice each (Figure 1(b)). Group 1 was control group, and groups 2–5 were administered 5 mg·kg^−1^ scopolamine hydrobromide (Sigma, St. Louis, MO, USA) intraperitoneally to induce memory impairment [10, 11]. Groups 1 (control) and 2 (model) received ddH2O intragastrically. Groups 3–5 were administered by gavage 0.1 mL per 10 g body weight of 0.3 g·kg^−1^ CSGNL (CSGNL-L), 0.6 g·kg^−1^ CSGNL (CSGNL-M), and CSGNL 1.2 g·kg^−1^ (CSGNL-H), respectively, once daily for 30 consecutive days. The CSGNL doses were chosen based on the orthogonal experiments of the optimal dosing ratios of CSGNL. Accordingly, a lower dose (0.6 g·kg^−1^) and a higher dose (1.2 g·kg^−1^) were used for the CSGNL-L and CSGNL-H groups. Ten minutes before the behavioral tests, mice in group 1 were administered normal saline intraperitoneally, and mice in other groups were administered scopolamine.

#### 2.6.2. Mouse Grouping for Morris Water Maze Test

Total 90 mice were divided into five groups with 18 mice each. In each group, 15 mice were used for Morris water maze test and the left 3 mice were used for hematoxylin and eosin (H&E) staining (Figure 1(c)). Group 1 was the control group, and groups 2–5 were administered scopolamine. Groups 1 (control) and 2 (model) received ddH_2_O intragastrically. Groups 3–5 were administered by gavage 0.3, 0.6, and 1.2 g·kg^−1^, respectively, once daily for 30 consecutive days.

### 2.7. Step-Through Test

The step-through test was conducted on days 29 and 30 after initiating intragastric administration (Figure 1(b)). Twenty minutes after intragastric administration of normal saline or CSGNL, scopolamine was injected to induce amnesia. The mice were placed in the bright room of a darkness-avoidance instrument (Chengdu Taimeng Technology, Chengdu, China) positioned with their backs toward a hole through which the mice could access the dark room. The time taken to enter the dark room was recorded as the latency. The mice were trained for 5 min and the number of errors for the mice made within 5 min was recorded. After 24 h, the same behavioral test was repeated and latency and errors within 5 min were recorded.

### 2.8. Morris Water Maze Test

The Morris water maze test was carried out daily from days 25 to 30 after the first intragastric administration using a mouse Morris water maze video tracking test system (WMT-100; Chengdu Taimeng Technology) (Figure 1(c)). Twenty minutes after intragastric administration of normal saline or CSGNL, scopolamine was administered. The temperature of water in the water maze was kept at 15–20°C and a platform was submerged 1 cm below the water surface. The mice were gently placed into the water pool, facing the pool wall. An electronic record was started immediately when the mice were in the water and was terminated when they found the platform. The mouse training time was set to 120 s and the time taken by the mice failing to reach the platform within 120 s was recorded as 120 s. Before the first test, the mice were placed near the platform and allowed to independently climb for 3 times. In the following tests, the mice were also placed near the platform but allowed to independently climb only once before each training. The time taken to locate the platform was recorded as the latency. Training was performed once every 24 h. During training, the mice were administered CSGNL and scopolamine once a day as described above. On day 30, the platform was removed from the water pool to assess spatial memory, as indicated by the platform residence time, the effective area residence time, the number of times that the mouse passed the platform, and the number of times that the mouse passed the effective area.

### 2.9. H&E Staining

Mouse brain tissues were fixed with 10% formalin, sectioned, and stained with hematoxylin and eosin stains (Figure 1(c)). Sections were photographed using an Olympus optical microscope (Japan).

### 2.10. AChE, ChAT, and ACh Assays

After the Morris water maze test, the mice were euthanized and brain tissues were removed immediately (Figure 1(c)). After washing and weighing, the brain tissues were homogenized in ice-cold normal saline with a mass ratio of 10% (w/v). Following centrifugation at 3000 rpm for 10 min at 4°C, the supernatant AChE and ChAT activities were assessed using commercial available kits (Nanjing Jiancheng Biological Engineering Institute, 20160421, Nanjing, China) and ACh content was assessed using an AChE ELISA kit (Groundwork Biotechnology Diagnosticate, 20160421, San Diego, CA, USA), read on an Infinite M200 microplate reader (Tecan, Switzerland).

### 2.11. Statistical Analysis

SPSS 19.0 (IBM SPSS Inc., Chicago, IL, USA) software was used for statistical analysis. Data were expressed as mean ± SEM. The differences among groups were compared by one-way ANOVA, followed by LSD method and Dunnett's post hoc test. Differences between the two groups were compared by Student's *t*-test. *p* < 0.05 was considered as statistical significance.

## 3. Results

### 3.1. HPLC Analysis of Active Components in CSGNL

**Figure 2:**
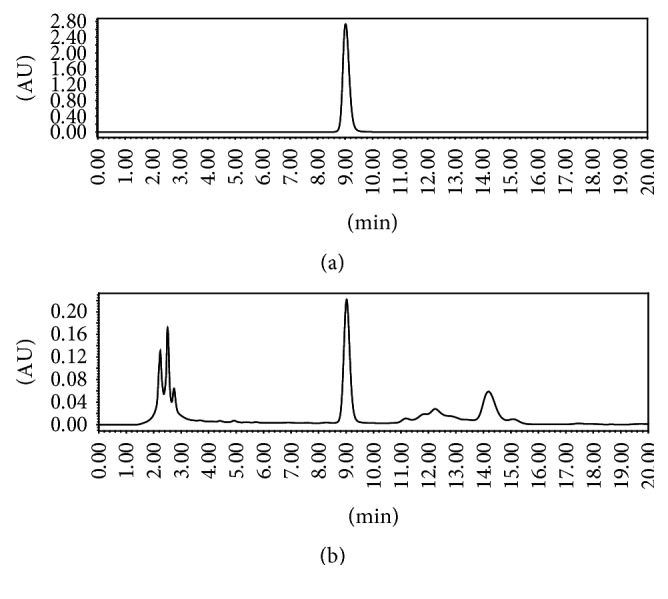
HPLC chromatograms of schisandrin. (a) Schisandrin reference compound. (b) Alcohol extract of* Schisandra*.

**Figure 3:**
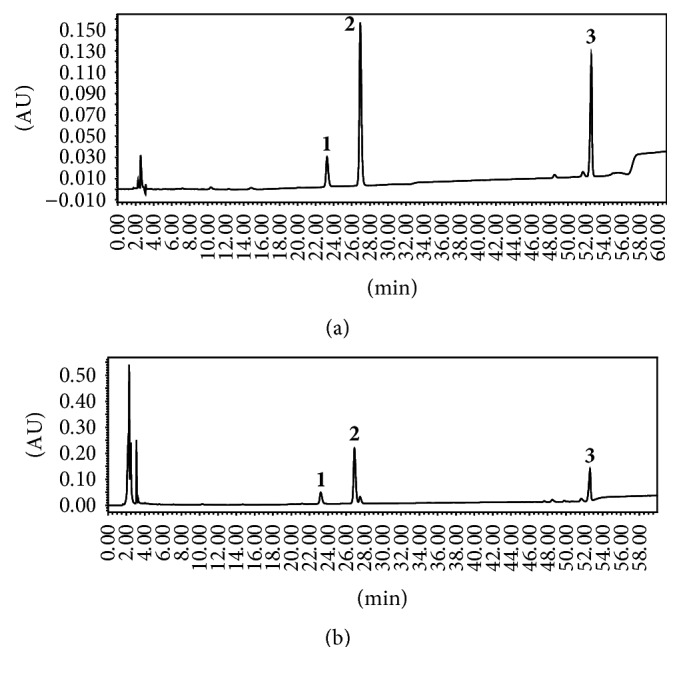
HPLC chromatograms of* Notoginseng*. (a) Reference compounds of notoginsenoside R1 (1), ginsenoside Rg1 (2), and ginsenoside Rb1 (3). (b) Tested active components in alcohol extract of* Notoginseng.*

**Figure 4:**
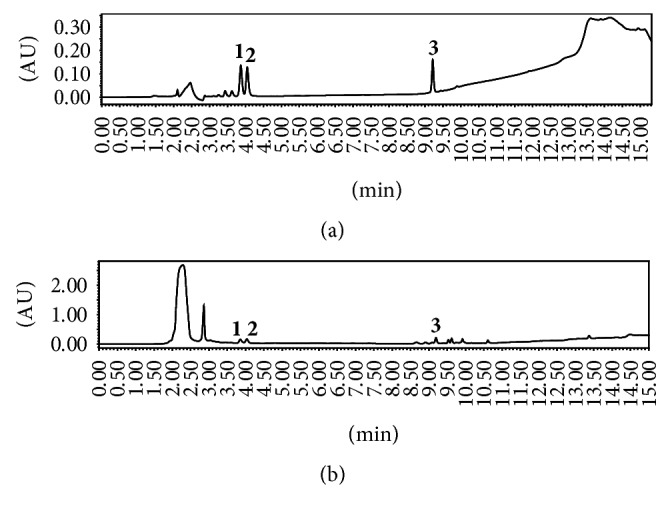
HPLC chromatograms of* Ginseng*. (a) Standard ginsenoside Re (1), Rg1 (2), and Rb1 (3). (b) Tested active components in alcohol extract of* Ginseng*.

The main active components of four herbal extracts were assessed using HPLC by comparing the retention time with respective reference compounds.  Figure 2 showed that 43.4 mg of schisandrin was extracted from 40 g of raw* Schisandra*. The active components of an alcohol extract of* Notoginseng* were shown in Figure 3. Total 146.0 mg notoginsenoside R1, 961.1 mg ginsenoside Rg1, and 312.4 mg ginsenoside Rb1 were isolated from 40 g crude* Notoginseng*.  Figure 4 showed HPLC chromatograms of* Ginseng*. About 8.4 mg ginsenoside Re, 8.5 mg ginsenoside Rg1, and 18.7 mg ginsenoside Rb1 were extracted from 40 g crude material. In addition, 1087.6 mg polysaccharides were extracted from 40 g crude* L. barbarum* materials measured by UV spectrophotometry.

### 3.2. CSGNL Dosing Ratio Optimization

**Figure 5:**
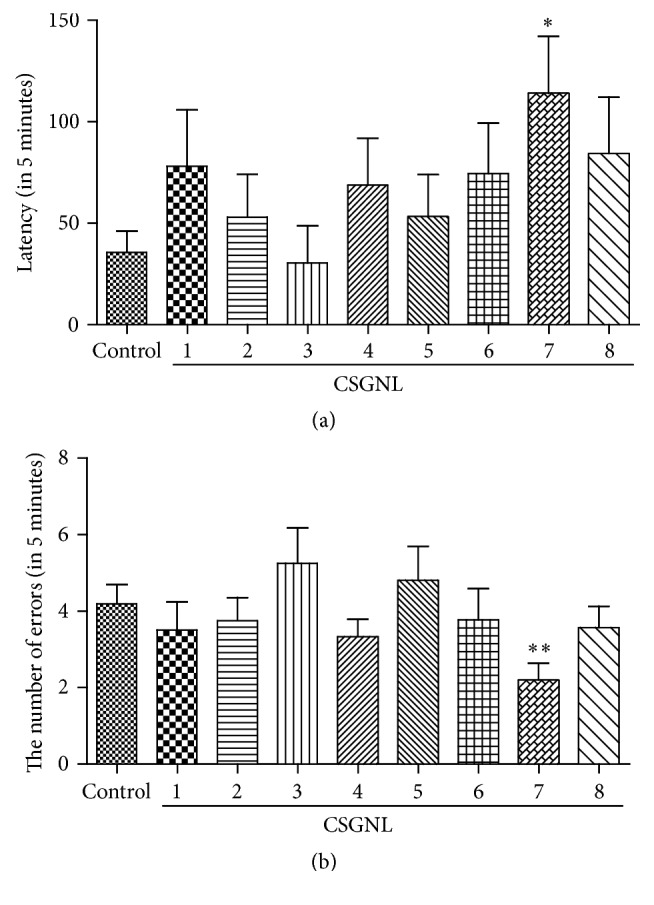
Effects of CSGNL in different dosing ratios on the learning and memory of mice in step-through test. (a) The latency. (b) The number of errors. The data are expressed as the mean ± SEM (*n* = 15). ^*∗*^*p* < 0.05 and ^*∗∗*^*p* < 0.01 versus the control group.

The optimal dosing ratio of the four herbal extracts was assessed using orthogonal experiments. A step-through test was applied to evaluate the effects of different ratios of CSGNL on learning and memory. The latency of mice was significantly longer (*p* < 0.05) and the number of errors was significantly lower (*p* < 0.01) in the CSGNL group 7 (A2B2C1D1) than in the control group (Figure 5), suggesting that the CSGNL group 7 dosing regimen is optimal among all tested groups, with total 0.6 g·kg^−1^ extracts composed of 0.148 g·kg^−1^* Schisandra* (1 g crude materials), 0.201 g·kg^−1^* Ginseng* (1.5 g crude material), 0.011 g·kg^−1^* Notoginseng* (0.16 g crude material), and 0.24 g·kg^−1^* L. barbarum* (1 g crude material).

### 3.3. Effects of CSGNL on Learning and Memory Function in Step-Through Test

**Figure 6:**
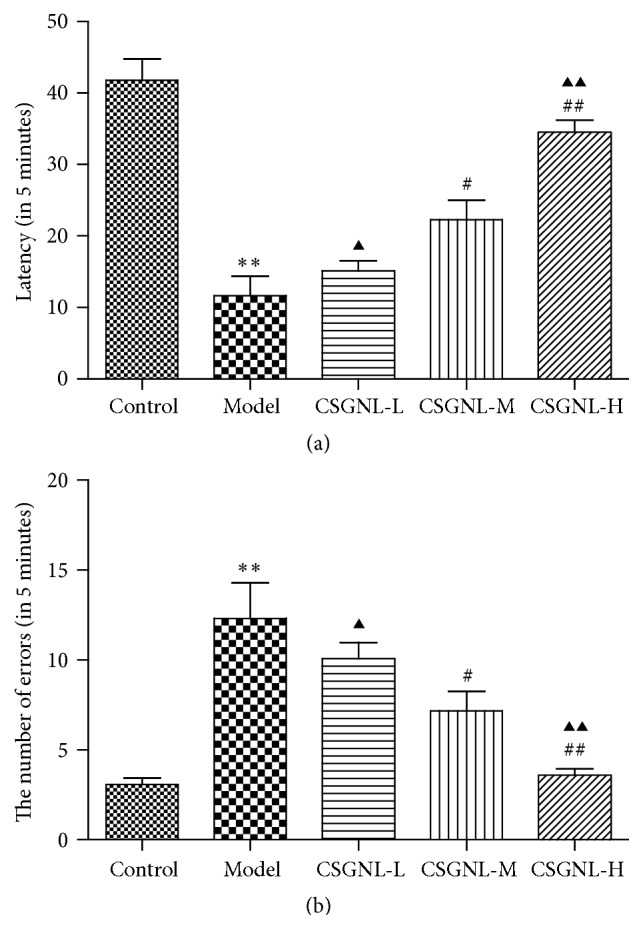
Effects of optimal CSGNL on the learning and memory of mice in step-through test. (a) The latency. (b) The number of errors. The data are expressed as the mean ± SEM (*n* = 15). ^*∗∗*^*p* < 0.01 versus the control group; ^#^*p* < 0.05 and ^##^*p* < 0.01 versus the model group; ^▲^*p* < 0.05 and ^▲▲^*p* < 0.01 versus the CSGNL-M group.

A step-through test was applied to evaluate the effects of the optimal CSGNL dose on the performance of mice with scopolamine-induced learning and memory impairment. The latency was significantly shorter (*p* < 0.01) and the number of errors made was significantly higher in the model group (*p* < 0.01) than the control group (Figure 6). However, amnesia induced by scopolamine was significantly improved in a dose-dependent manner by moderate (0.6 g·kg^−1^) and high (1.2 g·kg^−1^) doses of CSGNL, as indicated by longer latencies (*Ps *< 0.05) and fewer errors (*Ps *< 0.05), suggesting that CSGNL improved learning and memory function in scopolamine-treated mice.

### 3.4. Effects of CSGNL on the Learning and Memory Function in Morris Water Maze Test

**Figure 7:**
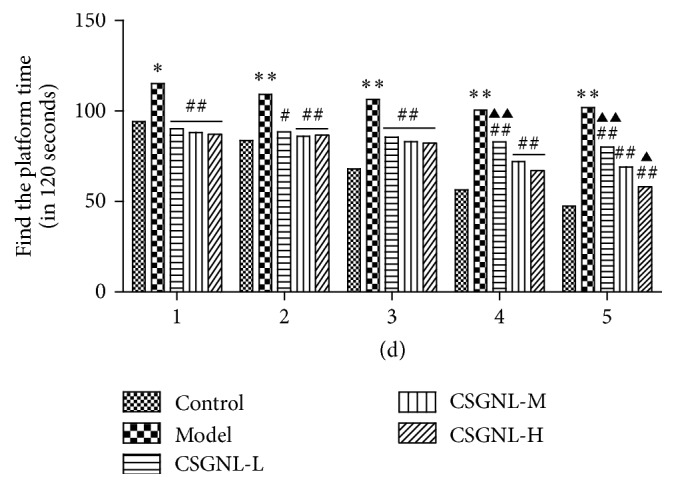
Effect of CSGNL on the latency throughout the 5-day training in Morris water maze test. The data are expressed as the mean ± SEM (*n* = 15). ^*∗*^*p* < 0.05 and ^*∗∗*^*p* < 0.01 versus the control group; ^#^*p* < 0.05 and ^##^*p* < 0.01 versus the model group; ^▲^*p* < 0.05 and ^▲▲^*p* < 0.01 versus the CSGNL-M group.

Morris water maze test was performed to evaluate the effect of CSGNL on learning and memory in scopolamine-treated mice. Compared to the control group, scopolamine-treated mice took longer time to locate the platform throughout the training course (Figure 7). However, all three doses of CSGNL significantly improved the performance of scopolamine-treated mice to find the platform, indicating that CSGNL ameliorated scopolamine-induced learning and memory impairment in mice.

**Figure 8:**
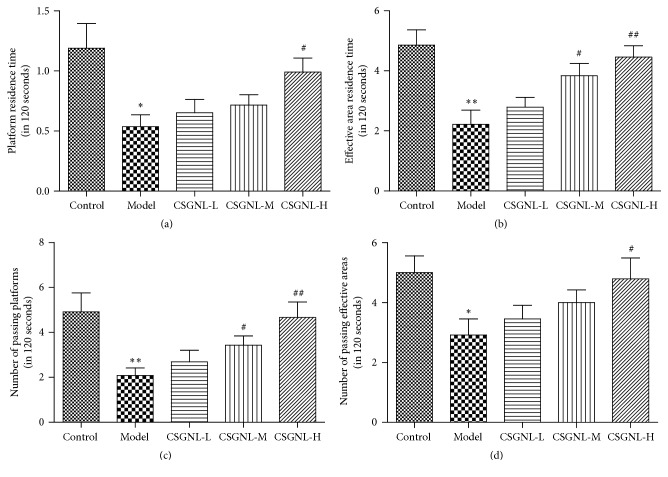
Effects of CSGNL on the spatial memory of mice in Morris water maze test. (a) Platform residence time. (b) Effective area residence time. (c) Time crossing the platform. (d) Time crossing the effective area. The data are expressed as the mean ± SEM (*n* = 15). ^*∗*^*p* < 0.05 and ^*∗∗*^*p* < 0.01 versus the control group; ^#^*p* < 0.05 and ^##^*p* < 0.01 versus the model group.

In the spatial memory test, scopolamine-treated mice spent significantly less time within the platform and effective area and crossed the effective area with significantly fewer times, compared to the control group (Figure 8). The moderate dose of CSGNL significantly increased both effective area residence time and platform crossing, and the high dose entirely ameliorated scopolamine-induced learning and memory impairment, further indicating that CSGNL ameliorated scopolamine-induced learning and memory impairment in mice.

### 3.5. Effects of CSGNL on the Pathological Changes of Hippocampus in Mice

**Figure 9:**
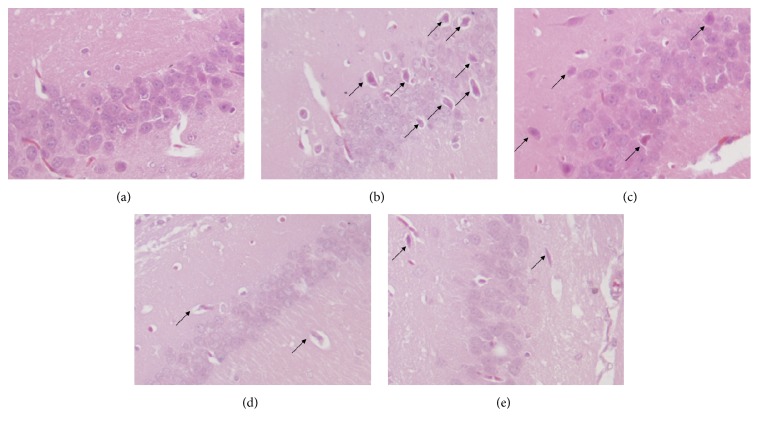
Effects of CSGNL on pathological changes of the hippocampus in the mice (200x). (a) Control group; (b) model group; (c) CSGNL-L group; (d) CSGNL-M group; and (e) CSGNL-H group.

Histopathological changes in the mouse hippocampus were examined by H&E staining (Figure 9). The neurons of the control group were intact, exhibited normal round nuclei, and were arranged in a neat and clear pattern. Neurons of the model group were shrunken with shrunk nuclei, H&E-stained cytoplasm, and loosely arranged pattern, exhibiting a typical morphology of degenerative or necrotic cells. The neurons of mice treated with a low dose of CSGNL did not significantly differ from those of the model mice; however, the neurons of mice administered moderate dose of CSGNL exhibited improved neuronal arrangement, and the neurons of those treated with high dose of CSGNL exhibited less neuronal degeneration and necrosis. These results indicate that CSGNL ameliorates scopolamine-induced damage to the hippocampus.

### 3.6. Effects of CSGNL on ChAT and AChE Activities and ACh Content of Mouse Brain Tissues

**Figure 10:**
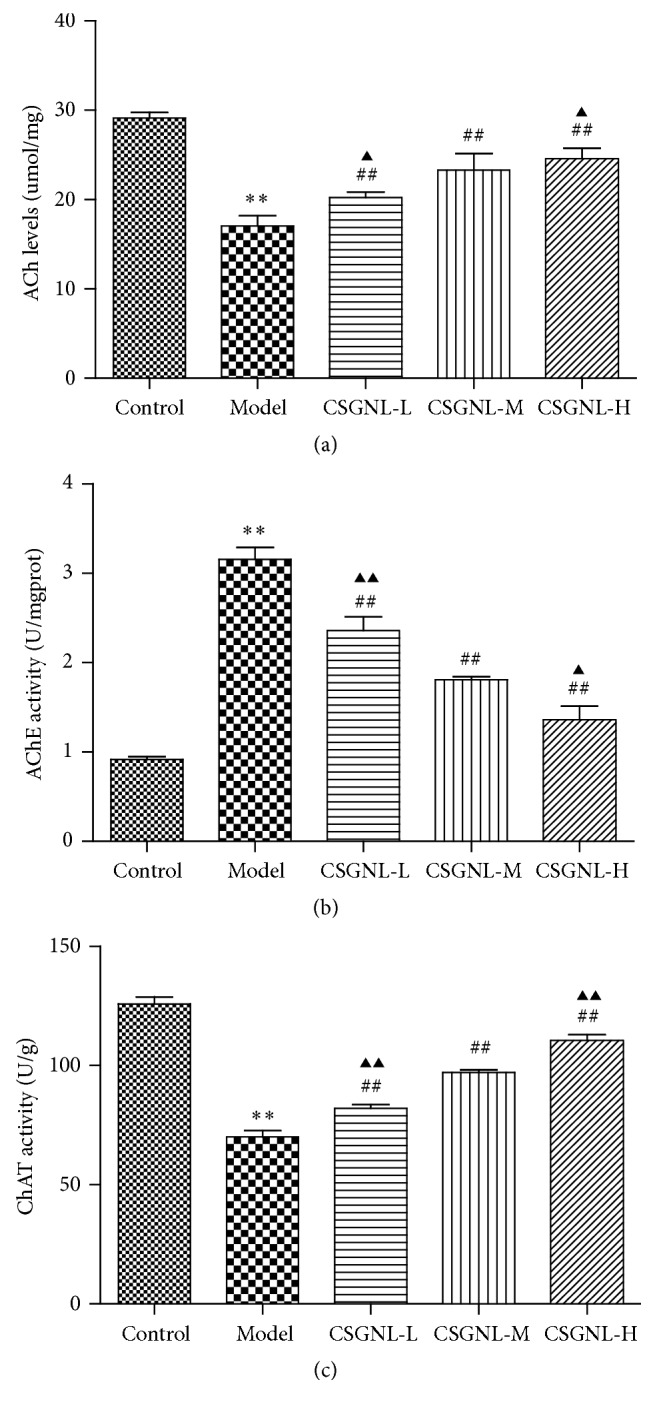
Effects of CSGNL on the ACh content and AChE and ChAT activities in mice brain tissue. (a) ACh levels. (b) AChE activity. (c) ChAT activity. ^*∗∗*^*p* < 0.01 versus the control group. ^##^*p* < 0.01 versus the model group. ^▲^*p* < 0.05 and ^▲▲^*p* < 0.01 versus the CSGNL-M group. The data are expressed as the mean ± SEM (*n* = 15).

AChE inhibitors were known to enhance cognitive abilities [12]; the effects of CSGNL on the ACh content and ChAT and AChE activities in mouse brain tissues were assessed. Scopolamine treatment significantly reduced the ACh level and ChAT activity but significantly increased AChE activity (*Ps *< 0.01) in the brain tissues of mice (Figure 10). However, all three tested doses of CSGNL significantly suppressed scopolamine-induced changes in the ACH levels and AChE and ChAT activities (*Ps *< 0.01) in a dose-dependent manner.

## 4. Discussion

Memory loss, also called amnesia, is common in the elderly. The purpose of this study is to develop a health food with an improving memory function. In line with the requirements by China Food and Drug Administration and based on the theory of traditional Chinese medicine, certain rules should be followed to prepare an herbal formulation. In general, at least two herbs need to be used in most Chinese health food formulations. It is believed that several traditional Chinese medicines in a formulation can interact with each other to enhance clinical effects. Traditional Chinese medicine practitioners believe that the brain is the location at which the marrow converges. When the marrow is sufficient, the brain will be nourished and allow learning and memory formation. The nourishment of brain is tightly associated with internal organs (i.e., heart, spleen, liver, lungs, and kidneys), in particular the kidney. The kidney essence is the main source of material to nourish the brain. When the essence is sufficient the marrow will be full, but if the essence is deficient the marrow will be empty. Therefore, reinforcing the kidney and supplementing the essence form the basis of treatment for memory loss in Chinese medicine. For the elderly, physical deficiency usually results in the symptoms of Qi and blood stasis, which can hinder the ability of the kidney essence to nourish the marrow. Modern pharmacological studies have demonstrated the health benefits of active phytochemicals tested in this study, including memory-improving activity.* Schisandra* lignans improved A*β* 1-42-induced short-term and spatial memory impairments and the memory impairment induced by cycloheximide in rats [13, 14]. Ginsenoside improved learning and memory and increased biosynthesis of the M-cholinergic receptor Bmax, cerebral proteins, and ACh [15]. Notoginsenoside increased synthesis of ACh by inhibiting AChE activity in the brain of rats, thereby improving learning and memory [16, 17].* L. barbarum* polysaccharides could inhibit various pathological indicators of Alzheimer's disease, such as reducing A*β* and glutamate toxicities, inhibiting Tau protein phosphorylation, and suppressing apoptosis [18, 19]. In this study, lignans of* Schisandra*, ginsenoside, notoginsenoside, and polysaccharides of* L. barbarum* were individually extracted from raw plant materials and combined into a candidate functional food, CSGNL, to improve learning and memory.

As a muscarinic antagonist, scopolamine can block acetylcholinergic receptors in the brain, thereby affecting memory generation and acquisition. Scopolamine impaired learning and memory in mice, which was consistent with previous observations [20–22]. Two distinct behavioral experiments, step-through test and Morris water maze test, were employed to robustly evaluate scopolamine-induced cognitive impairment. The step-through test or a passive avoidance test took advantage of mice's tendency to stay in the light and avoid darkness [23], and escape latency and error number were sensitive indexes of mouse memory processes [24]. The Morris water maze was a relatively objective evaluation recognized to measure learning and memory function [25, 26]. In the step-through test, mice treated with optimized CSGNL demonstrated prolonged latency and fewer errors than scopolamine-administered mice. Similarly in the Morris water maze, CSGNL reversed scopolamine-induced impairment of learning and memory. Both behavioral experiment results clearly indicate that CSGNL can alleviate learning and memory impairment induced by scopolamine in mice.

The hippocampus was part of the limbic system, an important brain region responsible for emotion, learning, and memory [27]. In this study, the histopathological changes observed in hippocampus tissues were evaluated by H&E staining. It was shown that CSGNL improved scopolamine-induced hippocampal damage.

Many neurophysiological and neurochemical mechanisms were involved in learning and memory, including in particular the cholinergic system [28, 29]. ACh was an important neurotransmitter required for normal cognition and memory, and ACh levels in the brain were significantly correlated with cognitive ability [30–32]. ACh metabolism in the brain depended on the activities of ChAT and AChE. ChAT catalyzed ACh synthesis, while AChE catalyzed ACh hydrolysis and degradation [33]. Higher ACh levels in the brain were associated with improved memory [34]. We observed that mice treated with CSGNL had higher ACh levels and ChAT activity, but lower AChE activity, than mice treated with scopolamine alone, suggesting that CSGNL improved learning and memory by increasing ACh levels via opposite regulation of AChE and ChAT activities.

In conclusion, CSGNL improves learning and memory in vivo by modulating the cholinergic nervous system. Our data support the new compound formula CSGNL as a promising functional food for enhancing learning and memory.

## Figures and Tables

**Table 1:** Extraction procedures of four herbs.

Medicine	Dosage (g)	Liquid-to-solid ratio	Solvent	Temperature	Time	Note
*Schisandra*	6	1 : 14	75% ethanol	65°C	2 h	—
*Ginseng*	9	1 : 10	80% ethanol	85°C	1 h	2 times
*Notoginseng*	1	1 : 8	50% ethanol	70°C	2 h	2 times
*Lycium*	6	1 : 10	Water	100°C	3 h	2 times

**Table 2:** Ratios of the mobile phase for the determination of ginsenosides by HPLC.

Time (min)	Acetonitrile (%)	Water (%)
0	30	70
5.5	36	64
12	80	20
20	100	0
25	30	70
30	30	70

**Table 3:** Ratios of the mobile phase for the determination of notoginsenosides by HPLC.

Time (min)	Acetonitrile (%)	Water (%)
0–12	19	81
12–60	19–36	81–64

**Table 4:** Four factors and two levels of orthogonal experiments (g·kg^−1^).

Level	Factor							
	A		B		C		D	
	*(Schisandra)*		*(Ginseng)*		*(Notoginseng)*		*(Lycium barbarum)*	
	Crude material	Extract	Crude material	Extract	Crude material	Extract	Crude material	Extract
1	0.3	0.044	0.5	0.067	0.16	0.011	1	0.24
2	1	0.148	1.5	0.201	0.5	0.068	2	0.48

**Table 5:** Optimization of the dosing ratios of orthogonal experiment groups.

Group	A	B	C	D
	*(Schisandra)*	*(Ginseng)*	*(Notoginseng)*	*(Lycium barbarum)*
1	1	1	1	1
2	1	1	1	2
3	1	2	2	1
4	1	2	2	2
5	2	1	2	1
6	2	1	2	2
7	2	2	1	1
8	2	2	1	2
